# GI Symptoms in Infants Are a Potential Target for Fermented Infant Milk Formulae: A Review

**DOI:** 10.3390/nu6093942

**Published:** 2014-09-25

**Authors:** Bert J. M. van de Heijning, Amelie Berton, Hetty Bouritius, Olivier Goulet

**Affiliations:** 1 Nutricia Research, Early Life Nutrition, P.O. Box 80141, 3508 TC Utrecht, The Netherlands; E-Mails: amelieberton@hotmail.com (A.B.); hetty.bouritius@danone.com (H.B.); 2 Department of Pediatric Gastroenterology-Hepatology and Nutrition, Necker Children’s Hospital, University of Paris, 75015 Paris, France; E-Mail: olivier.goulet@nck.aphp.fr

**Keywords:** infant milk formula, fermentation, GI function, GI symptoms

## Abstract

Besides pre- and pro-biotic-containing infant formulae, fermented infant formulae are commonly used to relieve or prevent symptoms of gastrointestinal (GI) discomfort in young infants. During the fermentation process in cow’s milk-based formulae, the beneficial bacteria modulate the product by forming several beneficial compounds, which contribute to the alleviation of the symptoms observed. This review summarizes the clinical evidence on the impact of fermented infant formulae on common pediatric GI-symptoms. The potential mechanisms involved are discussed: *i.e*., the lactose and protein (in-) digestibility, effects on gastric emptying and gut transit and modulation of the colonic microbiota. Although initial evidence indicates a beneficial effect of fermented formulae on GI discomfort in newborns, validation and confirmation of the clinical proof obtained so far is warranted, as well as further research to (more fully) understand the mode of action.

## 1. Introduction

Early in life, nutrition plays an important role in the development and functioning of both the gastrointestinal (GI) tract and the immune system. Transient GI disorders frequently occur in newborns along the course of their developmental path and might cause considerable discomfort to the infant and, hence, to its care givers. As the origin of the abdominal pain is often unclear, it cannot always be treated easily or adequately.

To alleviate persistent abdominal pain/discomfort in young children, special infant milk formulae have been manufactured and are readily available. Besides pre- and pro-biotic-containing infant formulae, fermented infant formulae may be a relevant alternative for the prevention or treatment of GI-related problems. The fermented milk formulae are produced by the fermentation of cow’s milk with specific strains of lactic acid bacteria, followed by mild heat treatment. Therefore, these formulae contain no viable bacteria, but only fermentation products.

This review focuses on fermented infant formulae and aims to provide an overview of the potential beneficial effects of fermented infant formulae on digestion and digestive health in young infants, to highlight their role in bringing relief and/or ailment of pediatric GI symptoms and to summarize what clinical evidence exists in this respect. Initially, a broad literature search was performed in which keywords related to fermented or acidified milk and infant nutrition were used. Additional sources were obtained from references in the reviewed articles or books. The language of publications used was limited to English and French.

We discuss and review the incidence of gastrointestinal discomfort in young infants (<6 months), the fermentation process and characteristics of fermented milk products, including a description of fermented infant formulae. Next, we evaluated the clinical use and evidence for beneficial effects of fermented formulae on digestion and/or digestive health and the potential mechanisms underlying those benefits. We conclude with some final remarks.

This review does not include the immunological effects *per se* of fermented formulae (see [1] for a recent review on this topic), although a healthy gut and, particularly, an intact barrier function of the intestinal wall are critically involved in the development and maturation of the (mucosal) immune system in the newborn [2]. Furthermore, digestive manifestations (spitting up, diarrhea and tummy aches, colic), which are the sequelae of immune-related issues (e.g., cow’s milk intolerance or allergy), are as such indistinguishable from GI symptoms with a different etiology.

In the present paper, we define “fermented infant formulae” in accordance with the European Society for Pediatric Gastroenterology, Hepatology and Nutrition (ESPGHAN) Committee on Nutrition, as “Infant and follow-on formulae that have been fermented with lactic acid-producing bacteria during the production process, but do not contain live bacteria in the final product due to inactivation of the fermenting bacteria by heat treatment or other means” [3].

### The Issue: GI Discomfort and Symptoms in Infants

Gastro-intestinal (GI) or digestive discomfort and disturbances comprise a variable combination of problems or symptoms, with different and mainly unknown etiologies, that prevail in otherwise healthy young infants and are sometimes also designated as “behavioral clinical conditions” (e.g., colic) [4]. Due to different criteria and methodologies used to diagnose these disorders, various and diverging incidence values are found in literature. The most relevant and recent data on GI discomfort frequency has been published by Iacono [5]: in this prospective, population-based study, the authors observed that 55% of healthy infants younger than six months suffer from at least one GI symptom. Although gestational age, birth weight and feeding habits seem to be the main parameters affecting these disorders, the etiology of the symptoms is not fully understood. Moreover, a survey among pediatric gastroenterologists on the “concept” of chronic abdominal pain in children revealed that no uniform or clear view, nor treatment, exists among clinicians on this most common symptom encountered in clinical practice [6,7]. Yet, the incidence of these inflictions keeps increasing and so do the medication- and non-medication-based treatments prescribed, despite substantially improved estimates of treatment responsiveness.

Although these GI problems are usually minor and not harmful for the baby, the problems can lead to feelings of distress for the parents or caregivers: infants can be inconsolable, and parents might no longer be able to cope and therefore seek medical help. Parental concern, rather than the patient’s symptoms, is the main reason for referral to a gastroenterologist [7]. Usually, GI problems resolve over time, and therefore, an effective nutritional solution is preferred over pharmaceutical treatment. One of the targets of a nutritional intervention might be the process of intestinal maturation, because it may be one of the underlying factors playing a role in symptoms of discomfort [8,9].

Regurgitation (reflux and spitting up), abdominal distension (bloating and ballooning), flatulence and colic are the most frequent GI disorders reported in young infants and are all considered as “minor” GI problems connected to the immaturity of the GI tract. The structure and function of the gut, as well as digestive enzyme production in the gut and pancreas still need to be fully activated [10,11]. Therefore, incompletely digested nutrients might enter the colon and be fermented by the (also developing) colonic microbiota, yielding excessive gas formation and causing problems (bloating). Furthermore, the valves and sphincters of the GI tract might not work properly yet; for instance, the lower esophageal sphincter preventing the stomach content from flowing back into the esophagus might still be leaky, leading to reflux and regurgitation.

Regurgitation is a frequently occurring GI problem seen more than once a day in 67% of healthy infants between 0 and 4 months [12] and is reported in around 23% of the population up to six months [5]. In the first year of life, as the infant grows, the esophagus’ length increases, and the immature lower esophageal sphincter gradually gets stronger; therefore, the chance of regurgitation reduces [13,14]. Spitting up after a meal, often after some delay, is also a GI manifestation of food allergy and, in its etiology, entirely different from reflux *per se*. Apart from scoring “visible” spitting, often by the caregivers, a more proper assessment of reflux is by esophageal pH monitoring and determining “the reflux index”: the percentage of time during which the esophageal luminal pH is below 4 or, likewise, by assessing the duration of the (longest) “reflux period” below pH 4 [15].

According to ESPGHAN, no dietary or therapeutic interventions are required to alleviate spitting and reflux, unless an underlying food allergy is suspected [14]. Postprandial spitting up and regurgitation of ingested milk might be prevented either by shortening the residence time of food in the stomach by increasing the gastric emptying rate and speeding up the pyloric passage of gastric content or by thickening the gastric content to impair esophageal reflux.

With a reported incidence up to 40%, colic is the second-most frequent GI disorder in young infants up to three months of age [4,5,16,17]. The peak of incidence is around six weeks of age [18,19] and in general resolves by the age of three months [20]. Colic is not fully understood, and several causative factors have been suggested, including from non-GI origin (e.g., an inadequate parent-infant interaction). However, most parents and many pediatricians consider this symptom to be linked to the GI tract: immaturity of gut function, food hypersensitivity or allergy and/or alterations in the gut microbiota are suggested to underlie this symptom [4,20]. In the dictionary, colic as such is defined as “a very spasmodic, griping pain in the belly”. As infants cannot verbally complain of colic (they cry), symptoms are designated as “colicky” when “full force crying in excess of 3 h per day, for 3 days or more per week” is observed, “together with spasm, lower limb flexure and diarrhea” [20,21,22]. In nutritional intervention studies, the reduction of crying time is often the measure of a beneficial effect on colic.

No clear data can be found in the literature on abdominal distension, ballooning and flatulence, even though these symptoms are also frequently reported in young infants by parents. Piemotese [23] reported an occurrence of 56% at eight weeks of age and still 36% at 16 weeks of age.

Although the majority of the gastro-intestinal discomfort symptoms described above has a favorable clinical course, is transient and disappears with age, the incidence is considerable. Given the distress when present for both the affected infants and their caregivers, prevention or treatment of GI symptoms is relevant and desirable. In an attempt to find a solution for colic and regurgitation, parents often switch formula [24].

## 2. A Solution: Fermented Infant Formulae

### 2.1. Fermentation

The fermentation process and the fermentation of food is a natural process used since ancient times. The initial and main role of fermentation was to preserve foods for longer periods of time. Fermentation has also frequently been used to generate foods with particular properties, palatability, taste and health benefits. A range of common food products, *etc.*, can be fermented (e.g., milk, meat, fish, cereals, fruits, *etc.*), generating products of large diversity (e.g., cheeses, wine, tea, bread, deli meats, condiments, *etc.*). The fermentation processes itself largely remained a mystery until the end of the 19th century, when the role of micro-organisms was discovered.

The fermentation process entails the anaerobic incubation (for a certain amount of time) of a food with specific strains of live bacteria. During this incubation, the bacteria grow and thrive on the food available and metabolize parts of it. The fermentation can be stopped by killing the bacteria, e.g., by controlled heat treatment. The fermented product may, hence, apart from the microbial metabolites, contain the live or killed bacteria, compounds and fragments thereof. It is noteworthy to differentiate the product fermentation, which occurred prior to ingestion, from the colonic fermentation of components from the same product that escape from digestion. Recently, the physiological properties of fermented milk ingredients (mainly peptides) were extensively reviewed by Beermann and Hartung [25].

Typically, in the case of the fermentation of milk (or milk-derived products), lactic acid-producing bacteria are used, and lactate is formed (mainly from lactose) during the fermentation process, rendering the fermented product more acidic and less susceptible to degradation and, hence, better preserved. Often, lactic acid-producing bacteria from the genera, *Lactobacillus*, *Streptococcus* and/or *Bifidobacterium*, are employed to ferment milk. Compared to the powerful lactic acid production during fermentation, only a small proteolytic activity toward the proteins in milk (casein and whey) is observed, and virtual no lipolysis occurs, as no lipases are available to hydrolyze the triglycerides in the milk fat globules. The milk carbohydrates (*i.e.*, mainly lactose) can also be metabolized into oligosaccharide (OS) strings (some with prebiotic action), e.g., galactose-OS (GOS) by transgalactosylation of lactose [26,27]. Therefore, in contrast to the carbohydrate (mainly lactose) moiety, the proteins and lipids in milk remain relatively untouched by the bacteria during the essentially anaerobic fermentation process. Because lactose is used as an energy source by the bacteria and converted mainly into lactic acid and GOS, fermented milk contains less lactose. However, in contrast to yogurt production, in most fermented formulae, the lactose decrement is insignificantly low (<3%).

The health benefits of fermented products and, more specifically, of fermented milk products are well established. Enhanced digestion and absorption have been linked to fermentation of milk products (e.g., for yogurts, see Adolfsson [28] and Boudraa [29]), but often, the mechanism behind the health benefits is not well understood, nor has it been possible in many cases to identify the “active” compound(s) involved. Nowadays, a lot of fermented milk products exist (e.g., yogurt, buttermilk, kefir, koumis, dahi), involving different microbial genera and processes, presenting different nutritional properties and constituting a significant part of the diet in many populations and at various ages: besides fermented milk products, also fermented infant formulae specifically designed for young infants are now available in some countries (e.g., in France).

### 2.2. Fermentation Products

Fermented milk products are dairy products obtained by fermentation of milk by the action of “GRAS”-micro-organisms (generally recognized as safe), *i.e.*, non-pathogenic, non-virulent, non-translocating. This results in a pH reduction with or without coagulation [30]. The coagulation refers to protein denaturation due to the acidification and the small, but vital, proteolysis occurring during the fermentation process. Yogurt is the best-known fermented milk product and is characterized by an acidic pH resulting from the fermentation of lactose by two specific lactic acid-bacteria starter cultures: *Streptococcus thermophilus* and *Lactobacillus bulgaricus* (or any *Lactobacillus* species as an alternative culture) [30]. Yogurt contains live microorganisms in the end product, and cultures must remain active at the end of the stated shelf life. Yogurt can hence be considered as a “probiotic” product, containing live bacteria, and displays health benefits linked to the presence of these live bacteria [31].

Fermented milk products that do not contain live bacteria should not be considered probiotic. Likewise, fermented milks also cannot be considered to be “prebiotic”, as no prebiotic oligosaccharides as such are added to the product. However, possibly metabolites of the killed bacteria are present in the milk that cannot be digested by the host and that might be used as a substrate by the commensal microbiota (e.g. oligosaccharides (OS), such as galactose-OS (GOS), fructose-OS (FOS) or *trans*-OS (TOS). Clinically relevant effects with respect to GI disorders of infant formulae containing either pro- and/or pre-biotics have been reviewed in depth elsewhere (Bragger [32], for term infants; Mugabe [33], for preterm infants). From now, we focus on the fermented infant formulae properties without elaborating on the pro-/pre-biotic properties *per se*. The impact on the residing microbiota, as was shown in healthy adults, is widely documented [34,35,36].

Next to the metabolites secreted by the live bacteria, also the cytosolic content (e.g., DNA and enzymes) and cell wall fragments (e.g., peptidoglycans) of the killed bacteria will be found in the fermented milk products. These compounds, although present in low concentrations in the fermented product, might exhibit functional properties often interacting with the host’s intestinal wall and/or commensal microbiota. The compounds or enzymes that are formed will depend on the bacterial strain employed, on the medium used (e.g., milk) and on the conditions during the fermentation process. Likewise, the functional and health effects of the fermented product depend on the fermentation process and the protocol applied. One of the characteristics of *Streptococcus thermophilus* and most lactobacilli*,* often used in the fermentation process, is the ability to produce the lactase enzyme, β-galactosidase, vital in converting the milk sugar, lactose, into lactic acid, thereby increasing the acidity and lowering the pH. By controlling the fermentation technology (e.g., choice of strains, inoculation size, pH course by aeration and stirring, incubation temperature and time), the characteristics of the fermentation end product can be monitored and should yield product parameters lying within a similar range each time (*i.e.*, should be reproducible). Fermentation protocols can entail co-incubation or sequential incubation of several bacterial strains and can be tailored by gauging incubation conditions to yield the desired characteristics and content of compounds (e.g., type and length of oligosaccharides) in the fermented product, *i.e.*, “functional fermentation” [37].

It is noteworthy that pasteurization of a fermented product not only kills the bacteria, but also drastically reduces the lactase activity. Pasteurized yogurt, hence, no longer results in an enhanced digestion of lactose, as is the case for fresh, unpasteurized yogurt. Nevertheless, according to some studies, pasteurized yogurt somehow retains its capacity to reduce symptoms of “lactose-intolerance” in children and adults [38,39,40]. Therefore, apart from the presence of lactase in the fermented product, other mechanisms and/or compounds might also be involved.

### 2.3. Fermented Infant Formulae

Fermented infant formulae, in accordance with legislation, not only meet the nutritional requirements of infants during the first months of life, but also present some additional specific characteristics, due to the fermentation process. One main characteristic is a low pH, due to the lactic acid content, and secondly, often the presence of lactase (*i.e.*, β-galactosidase). Many other metabolites may be present, as they might have appeared during the fermentation process. Some of them have been described, e.g., TOS (trans-oligosaccharide) was shown in infant formulae fermented with *Streptococcus thermophilus* and *Bifidobacterium breve* [26], but many others are not yet known. Thus, fermented formulae without live bacteria may indeed be considered to have “prebiotic effects” as such because their content of bacterial metabolites (some of which can be regarded as “prebiotics”) might be beneficial for the infant’s health [3].

## 3. The Evidence: Benefits of Fermented Formulae on GI Function in Infants

Although the benefits of fermented milk (and milk products) on health have been extensively studied and considered in many reviews, less seems to be known about fermented infant formulae. In 2007, The ESPGHAN Committee on Nutrition published a position paper on fermented infant formulae without live bacteria and reviewed the available clinical evidence in infants. Only two randomized controlled trials met their inclusion criteria, emphasizing the very limited amount of (reliable) data available [3]. The position paper cautiously concluded that there are indications (from one study only: Thibault [41]) that fermented formulae may reduce the severity of infectious diarrhea among healthy young infants. Based on the dropout rate and the adverse events reported, the committee (again with caution) did not raise any safety concern (with respect to growth) for the use of fermented formulae, although new studies were warranted.

### 3.1. History, the Early Experience

Fermented and/or acidified milks have been used in infant nutrition for a long time: sour milk was first introduced as a food for children in the late 1800s by Dutch farmers who fed their children buttermilk. At the beginning of the 20th century, enriched whole cow’s milk acidified by fermentation with lactic acid bacteria or by the addition of lactic acid has been used in infant nutrition in the case of severe malnutrition, premature birth or feeding anomalies. The objective of such a food product was to provide an easily digestible food of high caloric value. Several observational studies (by several pediatricians) performed at that time described “lactic acid milk” as being better tolerated by infants suffering from GI disturbances as compared to plain cow’s milk [42,43,44].

Several hypotheses were formulated to explain the observed increased tolerance and improved digestibility of lactic acid milk. Acidified milks were regarded to be more sterile and to have bactericidal properties. Particularly, the acidity was postulated to stimulate gastric and intestinal function and to inhibit pathogenic bacterial growth. In addition, the fineness of the curd was also assumed to have a positive effect on intestinal function or gastric emptying [43,45,46]. The methods of feed preparation (*i.e.*, by fermentation with lactic acid bacteria or by addition of lactic acid) were described as equivalent, as the beneficial effects were attributed to the acidification. Nevertheless, one must keep in mind that most of these assertions have been made on an empirical basis only (“what works”). Furthermore, hygiene and cleanliness in dairy industry was dubious and increased sharply since pasteurization and refrigeration was introduced [47].

The benefits of acidified milk were also questioned in a study with well-growing term babies in which no negative effects (*i.e.*, acidosis) were observed [46]. Furthermore, in premature babies, the acidified milk fed was shown to be inferior (*i.e.*, a lower weight gain) and to cause additional disturbances [45,48]. These perturbations were attributed to metabolic acidosis induced by lactic acid (or lactate), and in particular, the presence of d-lactate was questioned. l- and d-lactate are optical isomers that differ only in the position of the alpha-hydroxyl group. The predominant form (25:1) of lactate normally found in human blood and endogenously generated is l-lactate. The exogenous d-lactate molecule can be metabolized and cleared by the (still immature) kidneys, but in a much (30%) slower process compared to l-lactate. Hence, the presence of (high doses of) d-lactic acid may result in a metabolic acidosis, due to its accumulation in young infants, in particular in those infants with a significantly reduced (lactose) absorptive capacity in the small bowel [49]. Although no clear link has been established, since 1981, only non-pathogenic l-lactic acid producing bacterial cultures are permitted for the production of acidified formulae [50]. However, infants are still exposed to d-lactate, as bacteria from the genus *Lactobacillus* present in the resident colonic microbiota produce both lactate enantiomers. Hence, the caution that this might lead to acidosis in healthy infants is not warranted, and the restriction issued by the Codex can therefore be questioned [51,52].

Fermented/acidified infant formulae have been manufactured for more than 70 years now. These formulae, in accordance with the legislation, should, of course satisfy, the nutritional requirements of infants during the first months of life. In addition, as an extra benefit, these formulae may alleviate the symptoms in infants suffering from minor digestive discomforts of the lower gastro-intestinal tract, like colic, bloating and constipation. Indeed, early intervention studies concluded that fermented formula is associated with less diarrheal episodes and less vomiting in infants (e.g., [53]).

### 3.2. Clinical Evidence: Fermented Formulae and GI Function in Infants

Despite their widespread use over many years, only six randomized, controlled, double-blind studies [41,54,55,56,57,58] have been performed with fermented infant formulae. See Table 1 for an overview of recent (1989–2013) clinical studies with fermented infant formulae.

#### 3.2.1. Spitting Up and Reflux

Spitting up in general can be reduced by feeding smaller feeds, more frequently, and allowing the infant to properly burp up air during and after feeding: Swallowed air and gasses formed in the stomach can thus be released.

With regard to reflux treatment, currently, thickening of the stomach content is the most commonly applied dietary solution. Various thickeners can be added to formula (or expressed human milk) to increase the viscosity and have been clinically proven to alleviate reflux and regurgitation in infants under six months [59,60]. Thickeners added to a fermented formula are applied in the study by Roy and co-workers [54]: after 15 days, a fermented formula thickened with corn starch (with a high amylopectin content) was shown to decrease the frequency and severity of regurgitation, belching and hiccups in 47 infants aged 1–3 months. To what extent the fermentation and the presence of lactase in the formula contributed to these effects (on top of the added thickening starch) could not be determined.

Protein content and composition are essential for the gastric emptying rate: the more protein a milk contains, the slower its gastric emptying. A high casein content slows down the evacuation, due to curd formation in the acidic stomach milieu. The gastric emptying rate was tested by scintigraphically assessing the stomach content at 30 and 120 min after feeding in infants younger than 12 months with (*n* = 111) or without (*n* = 90) reflux complaints. Residual gastric activity 30 min after the meal was only 5% less in the group with reflux complaints compared to controls. At 120 min after feeding, in both groups of infants, irrespective of reflux symptoms, gastric residual radioactive content was 18%–22% when fed human milk, 25% in the case of acidified infant formula (“Pelargon”) and amounted to 38% for a standard formula [13]. The formulae used contained equal amounts of protein, predominantly casein (80%), and had a similar caloric density, osmolality and fat content, all known to affect the gastric emptying rate. Yet, the acidified formula, although casein-predominant, showed a gastric emptying similar to human milk (low in protein and whey-dominant), while a normal, casein-predominant formula had a lower gastric emptying at 120 min than human milk. Typically, gastric emptying rates depended on the type of milk used, irrespective of reflux complaints.

An acidified infant formula has a faster gastric emptying rate than a standard formula, and its gastric residence time does not differ from human milk. In the reflux group, gastric emptying was slightly more rapid, though highly variable. A slow gastric emptying rate should rather be considered as a possible aggravating factor in infants with reflux than a causative factor. It was concluded that acidified formulae may be used as an individual treatment for spitting up based on gastric emptying rates [13].

**Table 1:** Overview of clinical studies with fermented infant formula in the period 1989–2013.

Reference	Study Characteristics	Location	*N* (age)	Duration of Intervention	Population	Control Group Feeding	Intervention Group Feeding	Main GI-Related Results in the Fermented IF Group
Morisset *et al.* 2011 [56]	Multicenter, randomized, double-blind, controlled study	France	*N* = 129 (0–24 mo.)	Birth-12 mo.	Infants at high risk of atopy	Standard IF	Non-hydrolysed IF fermented by *Bifidobacterium breve* and *Streptococcus thermophilus*	√ Decreased GI symptom score at 4, 12 and 24 mo. (“digestive allergic manifestations”)
Campeotto *et al.* 2011 [55]	Multicenter, randomized, double-blind, controlled study	France	*N* = 58 (0–2 mo.)	During hospital stay: 2–5 wk	Pre-term infants (GA 30–35 wk)	Pre-term formula	IF fermented by *Bifidobacterium breve* and *Streptococcus thermophilus*	√ No effect on bacterial colonization √ Decreased fecal calprotectin in fermented formula group, significantly from Week 3 √ Lower incidence of abdominal distension
Garcette *et al.* 2007 [61]	Multicenter, longitudinal, observational study	France	*N* = 680 (1–3 mo.)	~30 d	Infants with digestive discomforts (bloating, gas, belching, unexplained crying)	NA	IF fermented by *Bifidobacterium breve* and *Streptococcus thermophilus*	√ Decreased symptom score during study period (e.g., bloating and gas 49 *vs*. 13%; unexplained crying 39 *vs.* 11%)
Indrio *et al.* 2007 [58]	Single center, randomized, double-blind, controlled study	Italy	*N* = 90 (0–4 mo.)	4 mo.	Healthy infants	Standard IF or Breast milk	IF fermented by *Bifidobacterium breve* and *Streptococcus thermophilus*	√ Fecal pH in intervention group was equal to breast-fed group, and lower than standard IF group
Roy *et al.* 2004 [54]	Multicenter, randomized, double-blind, controlled study	France	*N* = 109 (0–3 mo.)	15 d	Infants with digestive discomforts (unexplained crying, bloating, regurgitation, eructation, hiccups)	Standard IF	IF fermented by *Bifidobacterium breve* and *Streptococcus thermophilus*	√ Decreased intensity of digestive discomfort in intervention group √ Decreased gas in intervention group √ Tendency for decreased intestinal bloating
Mullié *et al.* 2004 [57]	Single center, randomized, double-blind, controlled study	France	*N* = 60 (0–5 mo.)	Birth-4 mo.	Healthy infants	Standard IF	IF fermented by *Bifidobacterium breve* and *Streptococcus thermophilus*	√ Increased fecal bifidobacterial level, significant at 4 mo.
Thibault *et al.* 2004 [41]	Multicenter, randomized, double-blind, controlled study	France	*N* = 971 (4–6 mo.)	5 mo.	Healthy infants	Standard IF	IF fermented by *Bifidobacterium breve* and *Streptococcus thermophilus*	√ Incidence and duration of diarrhea episodes are similar √ Less cases of dehydration (2.5% *vs.* 6%), less medical consultations (46% *vs.* 57%), less oral rehydration solution (ORS) prescriptions (42% *vs.* 52%) √ Fewer formula switches (60% *vs.* 75%)
Campeotto *et al.* 2004 [62]	Single center, open, prospective study	France	*N* = 69 (0–3 mo.)	3 mo.	Healthy newborns	Standard IF or Breast milk	IF fermented by *Bifidobacterium breve* and *Streptococcus thermophilus*	√ No GI symptoms scored √ No effect of the mode of feeding on fecal calprotectin concentrations in first week of life (d 3–7)
Romond *et al.* 1997 [63]	Single center, randomized, controlled study	France	*N* = 36 (0–15 d)	15 d	Healthy infants	Standard IF or Breast milk	IF fermented by *Bifidobacterium breve* and *Streptococcus thermophilus*	√ No GI symptoms scored √ Colonization of the intestinal microbiota in the fermented IF group is similar to the breast-fed group
Boudraa *et al.* 1994 [64]	Single center, randomized, controlled study	Algeria	*N* = 84 (0–5 mo.)	~3 mo.	Healthy infants	Standard IF	IF fermented by *Bifidobacterium breve* and *Streptococcus thermophilus*	√ Less infants with diarrhea √ Lower number of diarrhea episodes and with a shorter duration
Billeaud *et al.* 1990 [13]	Observational Study (with gastric emptying assessment)	France	*N* = 221 (<12 mo.)	NA	Healthy infants and infants suffering from gastro-esophageal reflux (GER)		Human or cow’s milk or various types of IF, among which an acidified milk: IF fermented with *Bifidobacterium breve* and *Streptococcus thermophilus*	√ Gastric emptying at 30 min and 120 min not different from human milk
Brunser *et al.* 1989 [65]	Multicenter, non-randomized, controlled study	Chile	*N* = 186 (<12 mo.)	6 mo.	Healthy infants	Standard IF	IF fermented by *Lactobacillus helveticus* and *Streptococcus thermophilus*	√ Lower number of episodes and duration of diarrhea √ Decreased carrier rate for enteropathogenic bacteria

GI, gastro-intestinal; IF, infant formula; GA, gestational age; mo., month; wk, week; d, day; NA, not applicable.

#### 3.2.2. Bloating and Abdominal Distension

In a multicenter, randomized, double-blind, controlled study, an infant formula containing 50% of fermented formula was investigated during two weeks in 0–3-month aged infants suffering from minor GI disorders. A decrease in intensity of the overall digestive discomfort was recorded for both groups studied, but the decrement was significantly larger in the fermented formula group [54]. More specifically, the decrease in “gas intensity” (flatulence) was significantly more pronounced in the fermented formula group; also, “intestinal bloating” tended to be lower in the fermented formula group. The latter result (on decreased bloating) was confirmed recently in a study with preterm infants, in which significantly more cases of abdominal distension were recorded in the standard formula group than in the group fed fermented formula ([55], see also below). Both studies cited used the same strains for fermentation (*i.e*., *Streptococcus thermophilus* and *Bifidobacterium breve*). These strains were also used in the study by Garcette and Bellaiche [61] on the outcome of a questionnaire (called “Serena”) on the use of a partially fermented (15%) formula in infants below four months of age. Although an improvement of the overall digestive comfort in infants suffering from minor GI discomfort was reported, no real conclusions can be drawn from this study, as there was no control group and as most of the GI disorders in young infants are disappearing with age anyway. Overall, a beneficial effect of fermented formulae on digestive discomfort can be observed.

#### 3.2.3. Diarrhea

The best investigated effect of a fermented infant formula is its effect on diarrheal disease in infants. Several studies [41,56,64,65] investigated the effects of fermented milk/formula in infants in the prevention or treatment of diarrheal disease. These studies indicate that infants may benefit from fermented infant formulas during diarrheal episodes. Thibault [41] studied the effect of a formula fermented by *Bifidobacterium breve* and *Streptococcus thermophilus* on the incidence of acute diarrhea in healthy 4–6-months-old infants in a randomized, double-blind, controlled trial. No effect on the incidence of diarrhea was observed, but severity indicators were affected: the number of dehydration cases, medical consultation and oral rehydration solution (ORS) prescription were less observed in the intervention group. In a separate study in infants at early weaning (<5 months of age), the same fermented infant formula has shown on top of lower occurrence, less severe and shorter periods of diarrhea [64].

A preventive effect has been observed in infants younger than 12 months of age (and of low socio-economic status) fed a formula fermented by *Lactobacillus helveticus* and *Streptococcus thermophilus* [65]. The incidence, the number of episodes and the duration of diarrheal episodes were lower in the experimental group when compared to the control group. The experimental group in this study also showed a decreased carrier rate for enteropathogens (*i.e.*, bacteria, rotavirus or parasites).

Moreover, a group of children at high risk of atopy were fed a non-hydrolysed, fermented formula for one year and were regularly monitored until two years of age [56]. The fermented formula reduced the adversive GI events by 50% at four months, even more so at 12 months (39% *vs.* 63%), and it tended to maintain the effect (*p* = 0.08) at 24 months, so well after the formula feeding was stopped. A working mechanism involving effects on the intestinal microbiota and the mucosal immune system was presumed, as suggested in earlier (pre-) clinical studies.

#### 3.2.4. Colic

Up to now, clinical studies that explicitly investigated the effects of fermented formulae on the prevention or treatment of colic are missing, but in some studies, related to lactose-intolerance, fermented milks are reported to affect crying time in colicky infants [21,22,66]. In two double-blind, placebo-controlled, crossover studies, formula had been pre-incubated with (yeast-derived) lactase drops for 24 h [21] or with (bacterial-derived) lactase drops for 4 h [22] prior to feeding. These two studies showed a significant reduction of the crying time and/or of the breath hydrogen excretion in colicky infants. Nevertheless, the small sample size of these trials (*n* = 13 in the Kearney study [21] and *n* = 32 compliant inclusions in the Kanabar study [22]) does not allow firm conclusions, as the lactose intolerance might also have been transient. Moreover, other earlier studies did not find these results: in a double-blind, crossover trial, Miller [66] reported no effect of added (yeast-derived) lactase drops either on the duration of the crying time or on the breath hydrogen excretion in breast-fed colicky infants. It is interesting to underline that in this small study by Miller (*n* = 15), the lactase drops have been given directly to the baby 5 min before each feeding. The authors mention the possibility that lactase (a protein) may have been inactivated/digested by the peptidases (pepsins) in the stomach. In the context of fermented milk, it is noted that bacteria do not need to be alive, but that intact cell walls are required to protect β-galactosidase/lactase activity duringgastric passage [67].

Colic may be related to bloating and problems due to gas formation in the colon [20], possibly due to a compromised or faulty digestion of lactose, as generally observed up to three months of age (“physiologic malabsorption”). Indeed, an elevated breath hydrogen excretion (indicative of a high bacterial metabolic activity) has been reported in colicky infants, suggesting malabsorption of lactose to be the cause of the colic [68].

Although two studies [21,22] suggest a positive effect of lactase pre-incubation in colicky infants, unclarity around the etiology of the colic persists, and the hypothesis of lactose malabsorption cannot explain all cases of the colic observed in small children. Nevertheless, fermented formulae may yield a better digestive tolerance and digestibility and be thus beneficial for infants in cases of colic related to lactose intolerance.

Very recently, we published the first data of a clinical trial in which a fermented formula combined with prebiotic oligosaccharides was shown to lower the incidence of colic in infants at four weeks of age by 60% [69].

#### 3.2.5. Colonization and Inflammation in Pre-Terms

Because of the lack of evidence on the safety of probiotics in pre-term infants [70], formula fermented by lactic acid bacteria (but without live bacteria in the final product) was regarded as an alternative option in this vulnerable population [55]. The effect of a formula fermented by *Bifidobacterium breve* and *Streptococcus thermophilus* was studied in pre-term infants (gestational age 30–35 weeks). The formula was well tolerated, and after two weeks of feeding, preterm infants in the fermented formula group had a reduced prevalence of abdominal distension and, probably linked to it, had significantly lower fecal calprotectin levels, suggesting that the fermented formula may have contributed in attenuating the inflammatory response. The microbiota composition in infants having fermented formula had a higher mean *Bifidobacteria* count, log_10_ 7.5 *vs.* log_10_ 6.6, than commonly found in term breastfed infants. The enrichment of *Bifidobacteria* correlates with a delayed colonization of the clostridial groups in infants born at a gestational age of >33 weeks. No effect of feeding was shown on the Enterobacteria or *Bacteroides* colonization. The authors concluded, based on the beneficial effects observed in this study [55], that feeding a fermented formula could be part of a strategy of minimal enteral feeding to accelerate the maturation of GI function in pre-terms.

## 4. Potential Mechanisms of Action: How?

The mechanisms by which fermented formulae reduce GI discomfort in infants are not completely understood. The underlying mechanisms could be associated with the improvement of the digestion process or the digestive tract health observed upon ingestion of fermented formulae. Table 2 summarizes the GI problems and their etiologies and how fermented formula might impact these disturbances.

### 4.1. Lactose and Lactase

The main carbohydrate in milk is the disaccharide, l-lactose; human milk contains about 7 g/dL lactose. As milk is the only and single source of nutrition for most mammalian and human sucklings and as lactose provides about 40% of the energy required by the newborn infant, the proper digestion and absorption of lactose are vital for the suckling’s growth and development. To this end, the enzyme, lactase (a β-galactosidase), is expressed and present predominantly in the newborn’s small intestine, embedded in the brush border membrane of the enterocytes lining its villi. In most mammals, the lactase expression is programmed to be transient and to disappear at weaning, as normally afterwards, lactose will be absent from the diet [71,72]. Lactase hydrolyses lactose into its constituent (l-anomeric) monomers, glucose and galactose, optimally at pH 6 and 25 °C, which can then be absorbed from the intestinal lumen.

Likewise, lactose can also be digested by some bacterial strains (e.g., the lactic acid-producing bacteria) that express lactase enzyme. Incubation, preferably anaerobic, of milk with these microorganisms, *i.e.*, fermentation, yields fermented milk (product) with an increased acidity, as (part of) the lactose was converted by the bacteria into lactic acid [28].

Incomplete lactose digestion in breastfed babies is normal and natural: up to 40% of the lactose in human milk is normally maldigested [47,73]; the stool characteristics (a greenish and aromatic paste) allow one to differentiate a breastfed from a formula-fed infant. The undigested lactose acts as a “prebiotic” in this case, but normally does not cause adverse effects. However, acute lactose maldigestion might lead to GI problems.

**Table 2:** Problems in a maturing GI tract, their presumed aetiologies and potential beneficial actions of fermented infant formulae.

GI Disorder		Presumed Aetiology		Potential Beneficial Action of Fermented Infant Formulae			References
Reflux and regurgitation		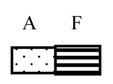		Less vomiting Faster gastric emptying Thickening gastric content			[53] [13] [54]
Bloating and ballooning		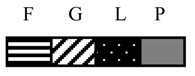		Less bloating Lactase addition Protease inhibition Microbiotal modifications			[54,55] [54,55,74,75] [25,76,77,78] [55,56,57,58]
Flatulence		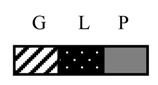		Less gas produced Lactase addition Protease inhibition			[54,55] [54,55,74,75] [25,76,77,78]
Colics		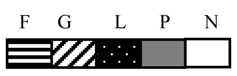		Reduced crying time Lactase addition Protease inhibition Microbiotal modifications			[21,22] [21,22,66,68] [25,76,77,78] [55,57,58]
Diarrhoea		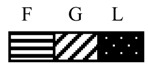		Less severe diarrhea Lactase addition Microbiotal modifications Anti-inflammatory metabolites			[41,49,53,64,65] [54,55,74,75] [34,56,57,58,63,79] [25,80,81]
				
Aetiologies	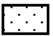		A: anatomical immaturity;		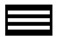	F: food hypersensitivity & allergy;	
	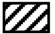		G: gut microbiota alterations;		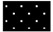	L: lactose maldigestion;	
	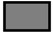		P: protein maldigestion;		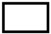	N: non-GI factors	
				

Diminished or too low lactase expression causes lactose intolerance (hypolactasia) and might lead to GI problems when lactose is ingested: the incompletely digested lactose might enter the colon and be fermented by the residing colonic microbiota, yielding excessive gas formation (mainly short-chain fatty acids (SCFA) are formed), leading to symptoms like abdominal distension, bloating, flatulence and/or colic. Upon association of these fatty acids with electrolytes, when produced in excess of the mucosal uptake capacity, they impose an extra osmotic load leading to watery stools and diarrhea. Other volatile by-products of lactose fermentation are methane, hydrogen and carbon dioxide. The majority leaves the body as flatus. Upon absorption by the mucosa and diffusion into the circulation, the hydrogen exhaled via the lungs is an indirect, but detectable indication used to quantify lactose maldigestion, although quite high lactose doses are commonly used for this test [47].

To solve the difficulties in lactose digestion, infant formulae, low or completely devoid of lactose, have been designed. However, long-term avoidance of lactose is not wise, as lactose is a key nutrient for young infants. Apart from being a fuel, lactose also facilitates Ca and Mg absorption [82] and is a source of galactose. In the early 1960s, it was suggested that galactose plays a role in the myelination process during brain development [83]. However, more recent reports on this topic are lacking.

Another solution is to keep the lactose in, but to provide exogenous lactase as present in fermented milk products or fermented formulae. Indeed, consumption of fermented milk products was shown to considerably alleviate GI symptoms (abdominal pain, diarrhea, flatulence) linked to the colonic fermentation of undigested lactose [68,84,85]. The lactase enzyme produced by *Streptococcus* *thermophilus* or most *Lactobacillus bulgaricus* strains during the fermentation process resists gastric stress and is also postulated to be one of the main engines responsible for the beneficial health effect of fermented infant formulae, particularly regarding the effect observed on symptoms linked to the distal digestive tract, *i.e.*, the colon [54].

Lactose intolerance is of less relevance for young infants, and congenital lactase deficiency is extremely rare [73]. However, particularly during the first two months of life, infants may not express sufficient lactase to digest the high lactose content of milk or formula [73]. Furthermore, during severe diarrhea, the intestinal epithelium might get damaged and lead to (secondary) lactose intolerance, as lactase expression in the small intestine is confined to the enterocytes’ villi. Indeed, 10%–20% of infants under two years presenting with diarrheal episodes show lactose intolerance, often in combination with cow’s milk allergy. Only a few studies have been performed in this respect in infants under 4–6 months of age (when only milk is given): a cautious and temporary use of lactose-free formula (or hydrolysates) can be considered in those cases of severe and persistent diarrhea [86].

In lactose-intolerant and malnourished children, a skimmed milk fermented by *Lactobacillus bulgaricus* and *Streptococcus thermophilus*, spray-dried with a specific temperature condition to maintain its β-galactosidase activity, has been successfully used. In an open study, the lactose of the fermented powdered milk containing a high lactase activity was perfectly absorbed by these children, as shown by the lower hydrogen respiratory test scores compared to a non-fermented infant formula [74,75]. Please note that also the lactose content of the fermented product was probably lower than the unfermented control formula.

### 4.2. Gastric Emptying

Compared to milk, yogurt shows a slower gastric emptying, resulting in a more regular release of nutrients into the intestine, both in man [87,88] and miniature pigs [89]. The observed delayed gastric emptying of yogurt and fermented milk products in general was attributed to a difference in food consistency and viscosity between the two dairy products: as already pointed out long ago, acidified milk yields a curd with a finer consistency compared to sweet milk [45]. Curd formation in the stomach mainly pertains to casein precipitating in the acidic milieu; likewise, it is probably the casein that is responsible for the coagulation observed in fermented milk products. A delayed gastric emptying somehow mediated the improved lactose digestion observed with fermented milk products and to a lesser extent with pasteurized fermented milk products [88,89,90]. In contrast to this delay, the gastric emptying rate of an acidified formula in infants has been shown to be rapid and similar to human milk, whereas the gastric emptying of cow’s milk in this study is slower than in human milk [13]. The seemingly contrasting data prevent a general conclusion, and therefore, a proper assessment of the role of gastric emptying can only be done for specific products and target populations.

### 4.3. Protein Digestion

The kinetics of the absorption of “nitrogenous compounds” present in milk and yogurt has been studied in man and pigs [88,89], and the results showed that both milk and yogurt proteins are highly digestible. Despite the slower gastric emptying (see previous paragraph), both dairy products were readily and almost completely (93%) absorbed. Fermentation was concluded not to affect the level of hydrolysis or the digestibility rate. Endogenous N secretion (enzymes) was similar, so no different bodily effort was observed in order to absorb the dietary protein from either dairy source.

However, as the immature GI tract in the newborn cannot readily digest and absorb all macronutrients adequately, residual proteins might enter the colon to be fermented by the locally-residing microbiota, thus leading to painful GI problems, like bloating and ballooning, due to the accumulation of volatile by-products of this fermentation [91]. Some of these GI symptoms, related to protein digestion, might be the first clinical manifestations of allergy and/or atopic disease. Hence, a hypoallergenic formula containing hydrolysed proteins may be an effective treatment. However, most infants with GI problems are not allergic and do not need to avoid intact proteins [4].

A formula with more easily and completely digestible proteins would be a better solution in this case. Preclinical data indeed show that fermented formulae have a more efficient protein digestion than standard formulae [76]. Without affecting their nutritional value, the fermentation and the resulting acidification of a formula might render the proteins more easily accessible for the proteolytic enzymes, probably due to conformational changes in the quaternary or 3D structure, as has been shown for whey proteins [92,93]. Furthermore, the bacterial proteolytic activity, albeit low, to which the proteins are exposed during the fermentation might contribute to these effect.

The hypothesis that fermentation changes the characteristics of proteins and makes them easier to digest has been tested in three-week old piglets, previously shown to be a valid model for the developing and maturing digestive tract of six-month-old human infants [94]. The piglets were fitted with an ileostomy and fed a non-fermented, standard infant formula or one consisting entirely of product (100%). In the group fed the 100% fermented formula, the ileal protein digestibility was higher than the control, and less protein (partially endogenous) would hence enter the colon [76,77]. Furthermore, a lower total proteolytic activity was measured in the ileal chymus compared to the standard formula group: activity levels of trypsin, chymotrypsin and elastase were shown to be much lower (~60% reduced). Apart from saving on the pancreatic secretory output in this way, a lower level of luminal proteases makes the luminal milieu less aggressive and harmful towards the enterocytes lining the gut. Proteases directly activate PARs (protease-activated receptors), shown in IBS patients to be associated with an increased sensitivity and pain perception and possibly triggering inflammation of the mucosal epithelium [95,96]. In addition to this, fermented formulae were shown to contain an increased protease inhibition capacity [78]. The fermentation of milk proteins has been reported to generate bioactive peptides among which Are protease inhibitors [25], helping to protect the gut wall.

With regard to the assumed conformational changes of the protein moiety of the formula, no assessments were made to compare fermented *vs.* unfermented product in this respect. More research and tool development is warranted in this emerging and potentially important field.

### 4.4. Modulation of Gut Microbiota

The impact of non-digestible oligosaccharides (prebiotics) and viable bacteria (probiotics) on the gut microbiota is widely documented in different age categories. Extremely relevant, but less documented is the very consistent effect of fermented infant formulae without live bacteria on the intestinal microbiota.

In healthy adults, who received for one week cell-free concentrated whey from *Bifidobacterium breve*-fermented milk or a formula fermented with *Bifidobacterium breve* and *Streptococcus thermophilus*, the fecal excretion of *Bacteroides fragilis*, *Clostridium perfringens* and clostridial spores decreases significantly. Bifidobacteria counts increased after consumption of cell-free concentrated whey from *Bifidobacterium breve*-fermented milk only. Fecal enzymes were also affected in this study [34]. Despite the absence of a control group in this study, which does not allow a firm conclusion, this study shows that the fermented product significantly affects the gut ecosystem microbial composition and functionalities.

Using the same strains, a fermented infant formula has also been investigated in newborn infants as compared to a standard formula and breast milk. The fermented formula seems to prevent the rapid colonization by *Enterobacter cloacae* in newborns [63,79]. Like in adults, the authors report that the fermented formula does not hamper the rapid development of *Bifidobacteria*.

Nevertheless, in the Mullié study [57], infants fed a formula fermented by *Bifidobacterium breve* and *Streptococcus thermophilus* had a higher mean proportion of *Bifidobacteria* at four months, and *Bifidobacterium longum*/*Bifidobacterium infantis* carriage was higher. In this study, the feces of infants up to four months of age were sampled monthly and analyzed for microbial content. Newborns fed a fermented formula from birth onward had a higher fecal proportion of *Bifidobacteria* than the infants fed a standard formula. Moreover, the *Bifidobacterium* species encountered in the fermented formula-fed group were similar as encountered in breast-fed infants, and those bacterial species were associated with an enhanced gut maturation and mucosal barrier function. This more breast milk-like microbial gut colonization is likely to connect to and to impact on the developing infant’s immune system in a beneficial way.

In a study in pre-term infants, no bifidogenic effect was found after two weeks on a formula fermented by *Bifidobacterium breve* and *Streptococcus thermophilus* [55]. It was concluded that the low level of *Bifidobacterium* at baseline, as measured in this study, does not allow the bifidogenic effect of prebiotic molecules.

Examining stool pH as an indicator of gut microbial metabolic activity, a result similar to Mullié [57] was found in a study with healthy term infants: the fecal pH of the group fed fermented formula (5.1) was lower than the group on standard formula (5.8), but similar to that of the breast-fed reference group (5.0) from the third postnatal day onward for the entire four months of the study [58]. Although neither the fermented formula nor the fecal microbiota were analyzed in this study, the authors suggest that “probiotic fermentation products” affected the microbial gut colonization process and were causative in the observed acidic shift in fecal pH. As no live bacteria are present in fermented formulae, but only “active metabolites” supporting the development of an optimal gut microbiota, which, in turn, might improve host immune responses; the authors also see an interest for the use of these formulae in premature infants.

In addition, Joosten and Lardeau [80,81] addressed another beneficial feature of the “active metabolites” or “probiotic fermentation products” present in fermented formulae: they show that these products are anti-inflammatory and, hence, might prevent the proliferation of pathogens (at least *in vitro*), thus reducing the neonatal exposure to these malign bacterial species. The beneficial effect observed in previous studies [41,56,64,65] on diarrhea prevention and its severity might be linked and/or explained by these bacteriostatic properties. As already stated above, the main active metabolite of fermented formulae (*i.e*., lactic acid) and the acidity also limit the possibility of bacteria growth *per se*. In rabbit pups challenged with *Enterobacter cloacae*, a biologically-acidified formula without live bacteria, a reduced incidence of gastric and pulmonary colonization compared to a standard or non-biologically-acidified formula was observed [97]. To date, a broad range of bacteriocins (anti-microbial peptides) produced during the fermentation process by lactic acid bacteria and present in fermented dairy products has been characterized. However, their (proteolytic) degradation during subsequent milk processing might restrict their beneficial anti-microbial activity for the consumer upon ingestion [25].

## 5. Conclusions

Minor GI problems are common in infants in the first six months of life. Hence, a formula specifically designed for infants with mild GI problems to solve these impositions might be advisable. As an alternative for standard formulae, fermented infant formulae were developed without live bacteria in the end product, but with metabolites having health promoting properties. Apart from meeting all nutritional requirements, as described in the EU directive, a somewhat lower lactose content, the presence of lactase, more easily digestible proteins, non-digestible oligosaccharides (TOS) and a low pH (<6) are the common features of fermented formulae.

It is important to state that unlike yogurts, fermented formulae are heat-treated and, therefore, do not contain viable bacteria. Clinical tolerance of fermented formulae was both good and similar in term and pre-term infants, with no adverse events reported. It may be concluded that these formulae might be suitable for use in premature infants, too.

Despite their widespread use over many years, the precise mode of action has not yet been elucidated conclusively. In this review, scientific substantiation for the health benefits of fermented formulae is given with respect to minor GI problems prevailing in infants up to six months of age, irrespective of their (possibly allergic) etiology. We focused on general GI problems, such as bloating, abdominal distension and spitting up and described the specific active compounds and/or characteristics of fermented formulae that could facilitate the alleviation/solution of these problems based on generic data. We described possible benefits and the underlying potential working mechanisms.

All specific active compounds and characteristics of the fermented product are naturally generated in a well-described and -controlled fermentation process and are the result of the metabolic activity of specific micro-organism used for this purpose since ancient times. The fermentation can be precisely monitored and controlled to yield highly reproducible products.

We had to restrict ourselves to the product characteristics as currently known: many other (possibly active) compounds, as yet unknown, that might arise during the fermentation step might be present in the product. Cracking the composition and nature of these compounds is a challenge for the future: the fermentation process and its resulting products can be even more directed and tailor-made with regard to their functional compound content in this way.

As discussed previously, fermented milks are commonly considered to be easily digestible. Even if a lot of studies, e.g., [13,85] have been conducted to investigate the digestibility of fermented milk as compared to human milk, still unknown areas persist. With respect to fermented infant formulae, to our knowledge, no clinical studies investigating the digestibility of such formulae in term or preterm infants have been performed or published.

GI problems consist of many different symptoms with multiple causes and can be treated in many different ways. As the etiology of the GI problems is mostly unknown, it is also difficult to select the optimal nutritional solution or, indeed, pharmaceutical treatment. Moreover, GI symptoms are often linked to each other, and therefore, combining active ingredients and the right formula characteristics in one treatment, a broader range of symptoms can be addressed and might be the most effective way to alleviate the prevailing GI problems. Fermented infant formulae are an interesting and promising option in this respect, and in the near future, the data of ongoing clinical trials are eagerly awaited to further substantiate the targeted benefits.

## References

[1] Granier A., Goulet O., Hoarau C. (2013). Fermentation products: Immunological effects on human and animal models. Pediatr. Res..

[2] Ruemmele F.M., Bier D., Marteau P., Rechkemmer G., Bourdet-Sicard R., Walker W.A., Goulet O. (2009). Clinical evidence for immunomodulatory effects of probiotic bacteria. J. Pediatr. Gastroenterol. Nutr..

[3] Agostoni C., Goulet O., Kolacek S., Koletzko B., Moreno L., Puntis J., Rigo J., Shamir R., Szajewska H., Turck D. (2007). Fermented infant formulae without live bacteria. J. Pediatr. Gastroenterol. Nutr..

[4] Savino F. (2007). Focus on infantile colic. Acta Paediatr..

[5] Iacono G., Merolla R., D’Amico D., Bonci E., Cavataio F., di Prima L., Scalici C., Indinnimeo L., Averna M.R., Carroccio A. (2005). Gastrointestinal symptoms in infancy: A population-based prospective study. Dig. Liver Dis..

[6] Schurman J.V., Hunter H.L., Friesen C.A. (2010). Conceptualization and treatment of chronic abdominal pain in pediatric gastroenterology practice. J. Pediatr. Gastroenterol. Nutr..

[7] Rosh J.R. (2010). Recurrent abdominal pain and the pediatric gastroenterologist: How are we functioning?. J. Pediatr. Gastroenterol. Nutr..

[8] Weaver L.T., Laker M.F., Nelson R., Lucas A. (1987). Milk feeding and changes in intestinal permeability and morphology in the newborn. J. Pediatr. Gastroenterol. Nutr..

[9] Thompson F.M., Catto-Smith A.G., Moore D., Davidson G., Cummins A.G. (1998). Epithelial growth of the small intestine in human infants. J. Pediatr. Gastroenterol. Nutr..

[10] Neu J. (2007). Gastrointestinal maturation and implications for infant feeding. Early Hum. Dev..

[11] Hamosh M. (1996). Digestion in the newborn. Clinics Perinatol..

[12] Hyman P.E., Milla P.J., Benninga M.A., Davidson G.P., Fleisher D.F., Taminiau J. (2006). Childhood functional gastrointestinal disorders: Neonate/toddler. Gastroenterology.

[13] Billeaud C.J., Guillet J., Sandler B. (1990). Gastric emptying in infants with or without gastro-oesophageal reflux according to the type of milk. Eur. J. Clin. Nutr..

[14] Aggett P.J., Agostoni C., Goulet O., Hernell O., Koletzko B., Lafeber H.F., Michaelsen K.F., Milla P., Rigo J., Weaver L.T. (2002). Antireflux or antiregurgitation milk products for infants and young children: A commentary by the ESPGHAN Committee on Nutrition. J. Pediatr. Gastroenterol. Nutr..

[15] Rudolph C.D., Mazur L.J., Liptak G.S., Baker R.D., Boyle J.T., Coletti R.B., Gerson W.T., Werlin S.L. (2001). Guidelines for evaluation and treatment of gastroesophageal reflux in infants and children: Recommendations of the North American Society for Pediatric Gastroenterology and Nutrition. J. Pediatr. Gastroenterol. Nutr..

[16] Canivet C., Hagander B., Jakobsson I., Lanke J. (1996). Infantile colic—Less common than previously estimated?. Acta Paediatr..

[17] Lucassen P.L., Assendelft W.J., van Eijk J.T.M., Gubbels J.W., Douwes A.C., van Geldrop W.J. (2001). Systematic review of the occurrence of infantile colic in the community. Arch. Dis. Child.

[18] Brazelton T.B. (1962). Crying in infancy. Pediatrics.

[19] Hide D.W., Guyer B.M. (1982). Prevalence of infant colic. Arch. Dis. Child..

[20] Gupta S.K. (2002). Is colic a gastrointestinal disorder?. Curr. Opin. Pediatr..

[21] Kearney P.J., Malone A.J., Hayes T., Cole M., Hyland M. (1998). A trial of lactase in the management of infantile colic. J. Hum. Nutr. Diet..

[22] Kanabar D., Randhawa M., Clayton P. (2001). Improvement of symptoms in infant colic following reduction of lactose load with lactase. J. Hum. Nutr. Diet..

[23] Piemontese P., Gianni M.L., Braegger C.P., Chirio G., Gruber C., Riedler J., Arslanoglu S., van Stuijvenberg M., Boehm G., Jelinek J. (2011). Tolerance and safety evaluation in a large cohort of healthy infants fed an innovative prebiotic formula: A randomized controlled trial. PLoS One.

[24] Polack F.P., Khan N., Maisels M.J. (1999). Changing partners: The dance of infant formula changes. Clin. Pediatr..

[25] Beermann C., Hartung J. (2013). Physiological properties of milk ingredients released by fermentation. Food Funct..

[26] Smart J.B. (1991). Transferase reactions of the beta-galactosidase from *Streptococcus thermophilus*. Appl. Microbiol. Biotechnol..

[27] Perrin V.F.B., Parly J.P., Lecroix F., Ta C.D. (2000). Identification and synthesis of a trisaccharide produced from lactose by transgalactosylation. Carbohydr. Res..

[28] Adolfsson O., Meydani S.N., Russell R.M. (2004). Yogurt and gut function. Am. J. Clin. Nutr..

[29] Boudraa G., Benbouabdellah M., Hachelaf W., Boisset M., Desjeux J.F., Touhami M. (2001). Effect of feeding yogurt *versus* milk in children with acute diarrhea and carbohydrate malabsorption. J. Pediatr. Gastroenterol. Nutr..

[30] CODEX STAN 243-2003. Codex Alimentarius WHO/FAO. Standard for Fermented Milks. http://www.codexalimentarius.org/standards.

[31] Guarner F., Perdigon G., Corthier G., Salminen S., Koletzko B., Morelli L. (2005). Should yoghurt cultures be considered probiotic?. Br. J. Nutr..

[32] Braegger C., Chmielewska A., Decsi T., Kolacek S., Mihatsch W., Moreno L., Piescik M., Puntis J., Shamir R., Szajewski H. (2011). Supplementation of infant formula with probiotics and/or prebiotics: A systematic review and comment by the ESPGHAN committee on nutrition. J. Pediatr. Gastroenterol. Nutr..

[33] Mugambi M.N., Musekiwa A., Lombard M., Young T., Blaauw R. (2012). Probiotics, prebiotics infant formula use in preterm or low birth weight infants: a systematic review. Nutr. J..

[34] Romond M.B., Ais A., Guillemot F., Bououader R., Cortot A., Romond C. (1998). Cell-free whey from milk fermented with Bifidobacterium breve C50 used to modify the colonic microflora of healthy subjects. J. Dairy Sci..

[35] Tzortis G., Goulas A.K., Gibson G.R. (2005). Synthesis of prebiotic galactooligosaccharides using whole cells of a novel strain, Bifidobactrium bifidum NCIMB 41171. Appl. Microbiol. Biotechnol..

[36] Depeint F., Tzortis G., Vulevic J., I’Anson K., Gibson G.R. (2008). Prebiotic evaluation of a novel galactooligosaccharide mixture produced by the enzymatic activity of Bifidobactrium bifidum NCIMB 41171, in healthy humans: A randomized, double-blind, crossover, placebo-controlled intervention study. Am. J. Clin. Nutr..

[37] Hayes M., Stanton C., Fitzgerald G.F., Ross R.P. (2007). Putting microbes to work: Dairy fermentation, cell factories and bioactive peptides. Part II: Bioactive peptide functions. Biotechnol. J..

[38] Savaiano D.A., AbouElAnouar A., Smith D.E., Levitt M.D. (1984). Lactose malabsorption from yogurt, pasteurized yogurt, sweet acidophilus milk, and cultured milk in lactase-deficient individuals. Am. J. Clin. Nutr..

[39] Lerebours E., N’Djitoyap Ndam C., Lavoine A., Hellot M.F., Antoine J.M., Colin R. (1989). Yogurt and fermented-then-pasteurized milk: Effects of short-term and long-term ingestion on lactose absorption and mucosal lactase activity in lactase-deficient subjects. Am. J. Clin. Nutr..

[40] Shermak M.A., Saavedra J.M., Jackson T.L., Huang S.S., Bayless T.M., Perman J.A. (1995). Effect of yogurt on symptoms and kinetics of hydrogen production in lactose-malabsorbing children. Am. J. Clin. Nutr..

[41] Thibault H., Aubert-Jacquin C., Goulet O. (2004). Effects of long-term consumption of a fermented infant formula (with *Bifidobacterium breve* c50 and *Streptococcus thermophilus* 065) on acute diarrhea in healthy infants. J. Pediatr. Gastroenterol. Nutr..

[42] Marriott W. (1919). The artificial feeding of athreptic infants. JAMA.

[43] Marriott W., Davidson L.T. (1923). Acidified whole milk as a routine infant food. JAMA.

[44] Schwartz A.B. (1926). The Use of Lactic Acid Milk in Infant Feeding. Am. J. Nurs..

[45] Karelitz S., Schell N.B., Goldman H.I. (1959). Lactic acid milk in the feeding of premature infants. J. Pediatr..

[46] Ungari S., Donath A., Rossi E., de Muralt G. (1965). The influence of the acidification of milk on the acid base balance of full-term newborns. Z. Kinderheilk.

[47] Solomons N.W. (2002). Fermentation, fermented foods and lactose intolerance. Eur. J. Clin. Nutr..

[48] Goldman H.I., Karelitz S., Seifter E., Acs H., Schell N.B. (1961). Acidosis in premature infants due to lactic acid. Pediatrics.

[49] Mack D.R. (2004). d(−)-lactic acid-producing probiotics, d(−)-lactic acidosis and infants. Can. J. Gastroenterol..

[50] CODEX STAN 72-1981. Codex Alimentarius WHO/FAO. Standard for Infant Formula and Formulas for Special Medical Purposes Intended for Infants. http://www.codexalimentarius.org/standards.

[51] Connolly E., Lonnerdal B. (2004). d(−)-lactic acid-producing bacteria. Safe to use in infant formulas. Nutrafoods.

[52] Connolly E., Abrahamson T., Bjorksten B. (2005). Safety of d(−)-lactic acid producing bacteria in the human infant. J. Pediatr. Gastroenterol. Nutr..

[53] Beland C., Cossette Y. (1960). Acidified powdered milk as a routine food for infants. Laval Med..

[54] Roy P., Aubert-Jacquin C., Avart C., Gontier C. (2004). Benefits of a thickened infant formula with lactase activity in the management of benign digestive disorders in newborns. Arch. Pediatr..

[55] Campeotto F., Suau A., Kapel N., Magne F., Viallon V., Ferraris L., Waligora-Dupriet A.-J., Soulaies P., Leroux B., Kalach N. (2011). A fermented formula in pre-term infants: Clinical tolerance, gut microbiota, down-regulation of faecal calprotectin and up-regulation of faecal secretory IgA. Br. J. Nutr..

[56] Morisset M., Aubert-Jacquin C., Soulaines P., Moneret-Vautrin D.-A., Dupont C. (2011). A non-hydrolysed, fermented milk formula reduces digestive and respiratory events in infants at high risk of allergy. Eur. J. Clin. Nutr..

[57] Mullié C., Yazourh A., Thibault H., Odou M.-F., Singer E., Kalach N., Kremp O., Romond M.-B. (2004). Increased poliovirus-specific intestinal antibody response coincides with promotion of *Bifidobacterium longum-infantis* and *Bifidobacterium breve* in infants: A randomized, double-blind, placebo-controlled trial. Pediatr. Res..

[58] Indrio F., Ladiza G., Mautone A., Montagna O. (2007). Effect of a fermented formula on thymus size and stool pH in healthy term infants. Pediatr. Res..

[59] Chao H.-C., Vandenplas Y. (2007). Comparison of the effect of a cornstarch thickened formula and strenghtened regular formula on regurtation, gastric emptying and weight gain in infantile regurgitation. Dis. Esophagus.

[60] Moukarzel A.A., Abdelnour H., Akatcherian C. (2007). Effects of a prethickened formula on esophageal pH and gastric emptying in infants with GER. J. Clin. Gastroenterol..

[61] Garcette K., Bellaiche M. (2007). Serena questionnaire: Course of functional disturbances and quality of life of suckling infants on Bledilait 1. Med. Enfance.

[62] Campeotto F., Butel M.-J., Kalach N., Derrieux S., Aubert-Jacquin C., Bardot L., Francoual C., Dupont C., Kapel N. (2004). High faecal calprotectin concentrations in newborn infants. Arch. Dis. Child. Fetal Neonatal Ed..

[63] Romond M.B., Yazourh A., Leroux B., Romond C. (1997). Effect of an Infant Formula Fermented by Streptococcus thermophilus and Bifidobacterium breve on the Implantation of the Microflora in the at Term Born Neonate.

[64] Boudraa G., Boukhrelda M., deLempdes J.B.R., Blareau J.P., Touhami M. (1994). Effect of fermented infant formula on incidence of diarrhea at early weaning (Abstract 41). J. Pediatr. Gastroenterol. Nutr..

[65] Brunser O., Araya M., Espinoza J., Guesry P.R., Secretin M.C., Pacheco I. (1989). Effect of an acidified milk on diarrhoea and the carrier state in infants of low socio-economic stratum. Acta Paediatr. Scand..

[66] Miller J.J., McVeagh P., Fleet G.H., Petocz P., Brand J.C. (1990). Effect of yeast lactase enzyme on “colic” in infants fed human milk. J. Pediatr..

[67] De Vrese M., Stegelmann A., Richter B., Fenselau S., Laue C., Schrezenmeir J. (2001). Probiotics—Compensation for lactase insufficiency. Am. J. Clin. Nutr..

[68] Moore D.J., Robb T.A., Davidson G.P. (1988). Breath hydrogen response to milk containing lactose in colicky and noncolicky infants. J. Pediatr..

[69] Vandenplas Y., Bouritius H., Ludwig T., Huet F., Hourihane J. (2014). A novel infant formula, combining scGOS/lcFOS with a specific fermented infant formula, shows lower incidence of colic in infants at 4 weeks of age compared to control formulas. J. Pediatr. Gastroenterol. Nutr..

[70] Agostoni C., Buonocore G., Carnielli V.P., de Curtis M., Darmaun D., Decsi T., Domellof M., Embleton N.D., Fusch C., Genzel-Boroviczeny O. (2010). Enteral nutrient supply for preterm infants: commentary from the European Society of Paediatric Gastroenterology, Hepatology and Nutrition Committee on Nutrition. J. Pediatr. Gastroenterol. Nutr..

[71] Sebastio G., Villa M., Satori R., Guzzetta V., Poggi V., Auricchio S., Boll W., Mantei N., Semenza G. (1989). Control of lactase in human adult-type hypolactasia and in weaning rabbits and rats. Am. J. Hum. Genet..

[72] Montgomery R.K., Büller H.A., Rings E.H.H.M., Grand R.J. (1991). Lactose intolerance and the genetic regulation of intestinal lactase-phlorizin hydrolase. FASEB J..

[73] Heyman M. B. (2006). Lactose intolerance in infants, children, and adolescents. Pediatrics.

[74] Gendrel D., Dupont C., Richard-Lenoble D., Gendrel C., Chaussain M. (1990). Feeding lactose-intolerant children with a powdered fermented milk. J. Pediatr. Gastroenterol. Nutr..

[75] Gendrel D., Richard-Lenoble D., Dupont C., Gendrel C., Nardou M., Chaussain M. (1990). Use of a fermented powdered milk in malnourished or lactose intolerant children. Presse Med..

[76] Huybers S., Abrahamse E., Knol J., Alles M., Bouritius H., Ludwig T. (2011). Fermented infant milk formula has high efficacy of protein digestion. J. Pediatr. Gastroenterol. Nutr..

[77] Huybers S., Abrahamse E., Knol J., Alles M., Bouritius H., Ludwig T. (2011). A fermented infant milk formula reduces ileal proteolytic activity. Pediatr. Res..

[78] Renes I.B., Schepens M., Krumholz F., Lambert J., Huybers S., Van den Braak C., Bouritius H., Knol J., Ludwig T. (2013). Specific fermentation of infant milk formula increases its protease inhibition capacity. J. Pediatr. Gastroenterol. Nutr..

[79] Yazourh A., Mullié C., Romond C., Romond M.B. (2000). Probiotic effect on reproduction of intestinal flora of the infant. Arch. Pediatr..

[80] Joosten J., Lardeau A. (2004). Enhanced microbiological safety of acidified infant formulas tested *in vitro*. S. Afr. J. Clin. Nutr..

[81] Joosten J., Lardeau A. (2008). Comments on Fermented infant formula without live bacteria. J. Pediatr. Gastroenterol. Nutr..

[82] Ziegler E.E., Fomon S.J. (1983). Lactose enhances mineral absorption in infancy. J. Pediatr. Gastroenterol. Nutr..

[83] Varma S.N., Schwarz V., Naomi Simpson I.M. (1962). The role of dietary lactose in the synthesis of brain galactolipids. Biochem. J..

[84] Kolars J.C., Levitt M.D., Aouji M., Savaiano D.A. (1984). Yogurt—An autodigesting source of lactose. N. Engl. J. Med..

[85] Onwulata C.I., Rao D.R., Vankineni P. (1989). Relative efficiency of yogurt, sweet acidophilus milk, hydrolyzed-lactose milk, and a commercial lactase tablet in alleviating lactose maldigestion. Am. J. Clin. Nutr..

[86] Chouraqui J.-P., Michard-Lenoir A.-P. (2007). Feeding infants and young children with acute diarrhea. Arch. Pediatr..

[87] Mahe S., Marteau P., Huneau J.-F., Thuillier F., Tome D. (1994). Intestinal nitrogen and electrolyte movements following fermented milk ingestion in man. Br. J. Nutr..

[88] Gaudichon C., Mahe S., Roos N., Benamouzig R., Luengo C., Huneau J.-F., Sick H., Bouley C., Rautureau J., Tome D. (1995). Exogenous and endogenous nitrogen flow rates and level of protein hydrolysis in the human jejunum after [^15^N]milk and [^15^N]yoghurt ingestion. Br. J. Nutr..

[89] Gaudichon C., Roos N., Mahe S., Sick H., Bouley C., Tome D. (1994). Gastric emptying regulates the kinetics of nitrogen absorption from ^15^N-labeled milk and ^15^N-labeled yogurt in miniature pigs. J. Nutr..

[90] Marteau P., Flourie B., Pochart P., Chastang C., Desjeux J.-F., Rambaud J.-C. (1990). Effect of the microbial lactase (EC 3.2.1.23) activity in yoghurt on the intestinal absorption of lactose: An *in vivo* study in lactase-deficient humans. Br. J. Nutr..

[91] Cummings J.H., Hill M.J., Bone E.S., Branch W.J., Jenkins D.J.A. (1979). The effect of meat protein and dietary fiber on colonic function and metabolism. II. Bacterial metabolites in feces and urine. Am. J. Clin. Nutr..

[92] Walstra P., Wouters J.T.M., Geurts T.J. (2006). Dairy Science and Technology.

[93] Onwulata C.I., Huth P.J. (2008). Whey Processing, Functionality and Health Benefits.

[94] Darragh A.J., Moughan P.J. (1995). The three-week-old piglet as a model animal for studying protein digestion in human infants. J. Pediatr. Gastroenterol. Nutr..

[95] Gecse K., Roka R., Ferrier L., Leveque M., Eutamene H., Cartier C., Ait-Belgnaoui A., Rostoczy A., Izbeki F., Fioramonti J. (2008). Incrased faecal serine protease activity in diarrhoeic IBS patients: A colonic lumenal factor impairing colonic permeabilty and sensitivity. Gut.

[96] Barbara G., Cremon C. (2008). Serine proteases: New players in diarrhoea-predominant irritable bowel syndrome. Gut.

[97] Boneti C., Habib C.M., Keller J.E., Diaz J.A., Kokoska E.R., Jackson R.J., Smith S.D. (2009). Probiotic acidified formula in an animal model reduces pulmonary and gastric bacterial load. J. Pediatr. Surg..

